# Genetic diversity of lion populations in Kenya: Evaluating past management practices and recommendations for future conservation actions

**DOI:** 10.1111/eva.13676

**Published:** 2024-03-19

**Authors:** Mumbi Chege, Bobbie Sewalt, Francis Lesilau, Geert de Snoo, Bruce D. Patterson, Linus Kariuki, Moses Otiende, Patrick Omondi, Hans de Iongh, K. Vrieling, Laura D. Bertola

**Affiliations:** ^1^ Wildlife Research and Training Institute Naivasha Kenya; ^2^ Institute of Environmental Sciences CML Leiden University Leiden The Netherlands; ^3^ Institute of Biology IBL Leiden University Leiden The Netherlands; ^4^ Netherlands Institute of Ecology (NIOO‐KNAW) Wageningen The Netherlands; ^5^ Negaunee Integrative Research Center Field Museum of Natural History Chicago United States; ^6^ Kenya Wildlife Service Nairobi Kenya; ^7^ Department of Evolutionary Ecology University of Antwerp Antwerp Belgium; ^8^ Department Biology University of Antwerp Antwerp Belgium; ^9^ Department of Biology University of Copenhagen Copenhagen Denmark

**Keywords:** connectivity, conservation genetics, diversity, SNP, translocation

## Abstract

The decline of lions (*Panthera leo*) in Kenya has raised conservation concerns about their overall population health and long‐term survival. This study aimed to assess the genetic structure, differentiation and diversity of lion populations in the country, while considering the influence of past management practices. Using a lion‐specific Single Nucleotide Polymorphism (SNP) panel, we genotyped 171 individuals from 12 populations representative of areas with permanent lion presence. Our results revealed a distinct genetic pattern with pronounced population structure, confirmed a north‐south split and found no indication of inbreeding in any of the tested populations. Differentiation seems to be primarily driven by geographical barriers, human presence and climatic factors, but management practices may have also affected the observed patterns. Notably, the Tsavo population displayed evidence of admixture, perhaps attributable to its geographic location as a suture zone, vast size or past translocations, while the fenced populations of Lake Nakuru National Park and Solio Ranch exhibited reduced genetic diversity due to restricted natural dispersal. The Amboseli population had a high number of monomorphic loci likely reflecting a historical population decline. This illustrates that patterns of genetic diversity should be seen in the context of population histories and that future management decisions should take these insights into account. To address the conservation implications of our findings, we recommend prioritizing the maintenance of suitable habitats to facilitate population connectivity. Initiation of genetic restoration efforts and separately managing populations with unique evolutionary histories is crucial for preserving genetic diversity and promoting long‐term population viability.

## 1 INTRODUCTION

Throughout their range, lions (*Panthera leo*) are important for cultural, economic and ecological reasons as top predators in the ecosystems they inhabit (Sinclair, 2003; Wolf & Ripple, 2018). However, their population size has declined dramatically in recent decades due to anthropogenic activities (Bauer et al., 2015; Riggio et al., 2013). These activities have disrupted the once‐connected network of lion populations, resulting in a loss of approximately 50% of connected habitats within the eastern and southern African regions and a 65% population decline in East Africa in the last half century (Loveridge et al., 2022). Consequently, to safeguard the remaining lion populations and their habitats fencing of wildlife areas, although contentious, has increasingly become a popular conservation strategy (Creel et al., 2013; Lindsey et al., 2017; Packer et al., 2013).

Kenya hosts 2 of the 10 remaining lion strongholds in Africa (Riggio et al., 2013) and has an estimated population of 2400 lions (Kenya Wildlife Service, 2020). The Kenyan lion population is characterized by a meta‐population structure where lions are distributed across a fragmented network of formally protected (national parks, reserves and conservancies) as well as unprotected areas. The population shows a declining trend, attributed to human–lion conflicts that have been exacerbated by shrinking lion habitat (Bauer et al., 2005; Kenya Wildlife Service, 2020). These effects are reported to be more pronounced in unfenced and/or in unprotected areas, especially those close to human settlements (Dolrenry, 2013; Harcourt et al., 2001; Woodroffe, 2000).

Kenya Wildlife Service (KWS), a government institution with the mandate to conserve and protect wildlife in Kenya, employs fencing and translocation among other conflict mitigation methods (Kenya Wildlife Service, 2018). Consequently, several wildlife areas in Kenya are either partially or completely fenced to avoid conflicts (Kenya Wildlife Service, 2018). While fencing can play a role in reducing conflicts, it often results in isolation due to diminished habitat connectivity (Creel et al., 2013). This, in turn, hampers the natural movement and dispersal of lions, especially affecting small and isolated populations that are more vulnerable to environmental and demographic stochasticity (Miller & Funston, 2014). Further, these populations are at a higher risk of experiencing loss of genetic diversity and inbreeding due to genetic drift and reduced gene flow (Björklund, 2003; Curry et al., 2020; Frankham et al., 2019; Miller et al., 2015).

On the other hand, translocation in Kenya dates back to mid‐1950s, and target individuals include ‘problem’ lions—defined as an individual that kills more livestock per encounter than other individuals (Linnell et al., 1999) and involves ‘problem’ wild‐living individuals of either sex. Tsavo and Meru National Parks (NP) have long served as the main recipient sites to which ‘problem’ lions are translocated (Jenkins, 1996). Other conflict mitigation methods have included lethal control albeit being selectively employed, that is, by European settlers in the 1900s (in the Laikipia Plateau in central Kenya), it however led to extirpation of lions from most parts of Laikipia by the 1960s (Denney, 1972). By KWS in Aberdares NP between 1990 and 2000s as a response to over‐predation of the endangered Mountain Bongo (*Tragelaphus eurycerus isaaci*) and bushpig (*Potamochoerus larvatus*) (Massey, 2013); and in Lake Nakuru NP 37 individuals were euthanized in 2002 as a result of killing two rangers (Ogutu et al., 2012). However, lethal control is seen as a last resort and translocation remains the preferred conflict management method due to the endangered status of lions in Kenya (Kenya Wildlife Service, 2018).

To effectively manage lions in Kenya, it is important to ensure that the populations are stable and are not subjected to genetic erosion. Populations therefore ought to be managed both on the population and on the genetic level. In this context, it is important to acknowledge both historical events and past management interventions that may have influenced patterns of diversity observed today. As well, to ensure the long‐term viability of the lion populations, it is important to recognize the lion population genetic structure and diversity, and to ensure connectivity of core lion habitats that allow gene flow (Bertola, Sogbohossou, et al., 2022; Bertola, Vermaat, et al., 2022; Björklund, 2003; Miller et al., 2015). Indeed the national recovery and action plan for lions in Kenya 2020–2030, calls for improved understanding of lion population genetics and collection of baseline information on population structure to guide decision making (Kenya Wildlife Service, 2020).

However, only a handful of lion genetic studies have been undertaken in Kenya: a study using mitochondrial DNA found strong divergence between the southern and northern Kenya populations (Bertola, 2015) and another found a relatively high number of monomorphic microsatellite loci (3 out of 20) in the Amboseli NP population (Bertola et al., 2015), attributed to a colossal population decline in 1990 (Chardonnet, 2002). The population later recovered after the immigration of lions from surrounding areas (Dolrenry et al., 2014). This illustrates that genetic diversity can be rapidly lost in natural populations, making genetics an important consideration for lion management. As far as we know, there never has been a genetic assessment of the fenced or semi‐fenced lion populations in Kenya, even though these would be obvious candidates for genetic management. The overall lack of genetic baseline data in Kenya, and many other places in the world, hampers informing conservation policy and management actions with genetic data.

Therefore, this study aimed to create a genetic baseline for lion populations in Kenya and investigated population structure, differentiation between and genetic diversity within populations. Thus, the main objectives of our study were: (1) to investigate the distribution of genetic diversity of lions in Kenya, in the context of known population histories; (2) to assess the impact of management interventions, that is, fencing and translocations on local lion genetics and (3) to translate our insights from genetic data into recommendations regarding policy and management for lion conservation in Kenya. Following previous results, based on mitochondrial data only, we expected to find pronounced population structure along a north‐south gradient in Kenya. We further expected to find populations with very limited connectivity, for example, as a result of fencing, to show strong differentiation from other populations. If a population displays very low levels of genetic diversity, genetic rescue (i.e., the introduction of new alleles) specifically to boost population fitness may be warranted (Bertola, Sogbohossou, et al., 2022; Bertola, Vermaat, et al., 2022; Frankham, 2015). In this regard, the translocation of ‘problem’ lions may contribute by providing a source of animals to increase within‐population diversity by mimicking gene flow (Miller et al., 2013). The wildlife translocation protocol in Kenya has not yet taken into account the genetic properties of ‘problem’ lions being translocated or of the recipient population (Kenya Wildlife Service, 2019), and therefore, we expected to find signatures of past translocations in populations which have received ‘problem’ lions in the past. Finally, with these baseline data, future genetic monitoring could serve as an early warning system that allows managers to intervene and counteract the loss of diversity before negative fitness consequences become apparent (IUCN/SSC, 2013).

## 2 MATERIALS AND METHODS

### 2.1 Study sites and sample collection

**FIGURE 1:**
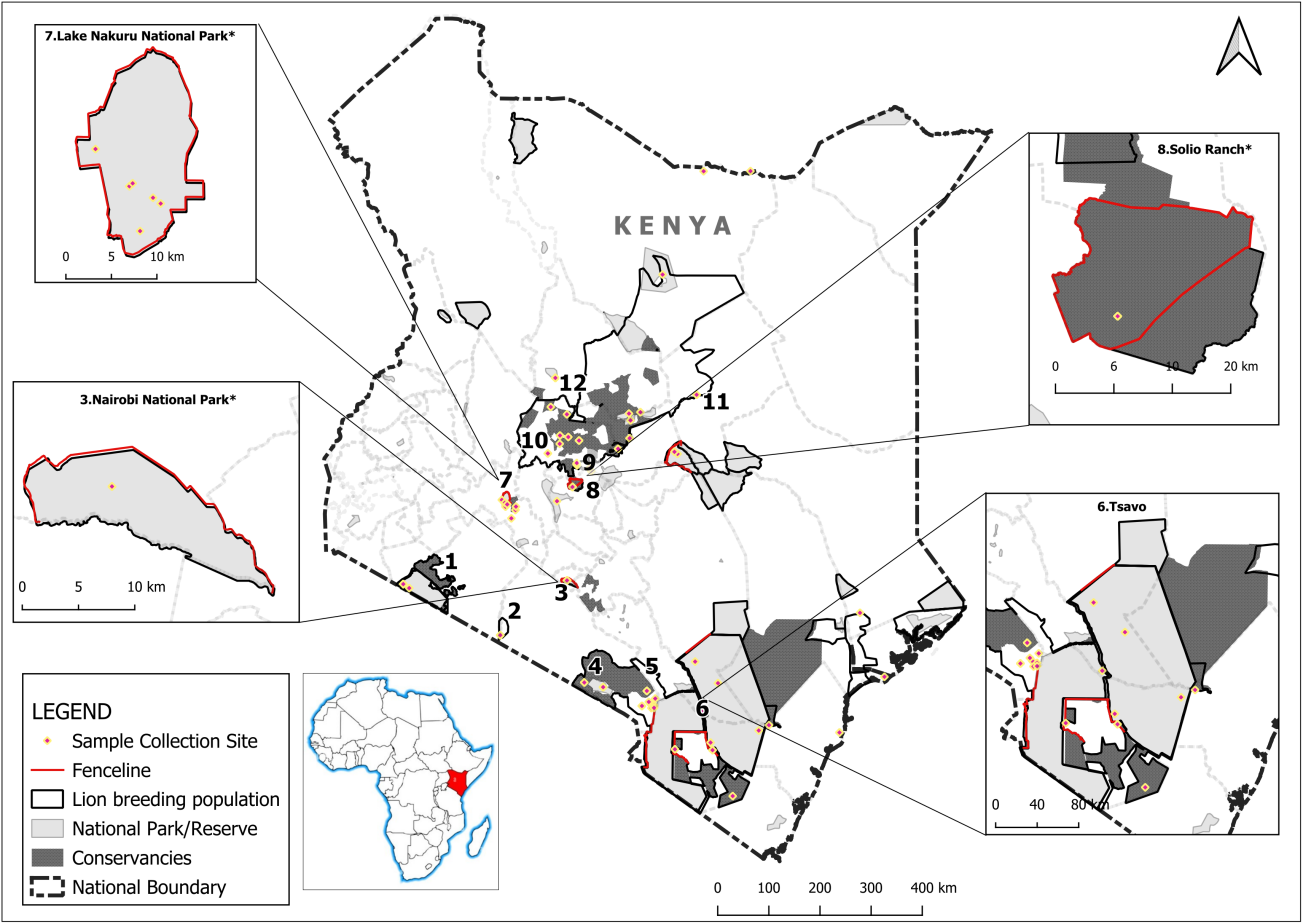
Map of Kenya highlighting the 12 lion populations. Red dots with a yellow outline show the sample collection sites. The four map excerpts display the fence lines (red lines). The *asterisk signifies the closed populations. The numbers correspond to 1—Maasai Mara; 2—Shompole, 3—Nairobi*, 4—Amboseli, 5—Chyulu Hills, 6—Tsavo, 7—Nakuru*, 8—Solio Ranch*, 9—Nanyuki, 10—Laikipia, 11—Isiolo and 12—Maralal.

A total of 171 lion blood, tissue and hair samples were acquired from Bertola, 2015, KWS depositories, opportunistically during collaring and were obtained from areas with permanent existence of lion populations (Figure 1)—all collected between 2010 and 2020. These areas comprise formally protected areas, that is, national parks, reserves and conservancies (i.e., Maasai Mara, Shompole, Nairobi, Amboseli, Chyulu Hills, Tsavo, Nakuru, Solio Ranch and Laikipia) and unprotected areas of Maralal and sections of Isiolo (Table 1).

The samples were preserved in a buffer solution (0.15 M NaCl, 0.05 M Tris–HCl, 0.001 M EDTA, pH 7.5) and were collected in full compliance with legally required permits obtained from KWS (export permits no. 0004289 and no. 0011543, import permits no. 17NL239882/11 and no. 21NL295377/11).

### 2.2 DNA isolation and SNP genotyping

For the samples which had not been previously processed at Leiden University, the Netherlands, DNA isolation was performed at the KWS genetics and forensics lab in Nairobi, using the DNeasy Blood and Tissue kit (Qiagen) following the standard protocol. Following extraction, Whole Genome Amplification (WGA) was performed on all samples using the WGA Kit from LGC genomics at the molecular lab of Leiden University. After WGA amplification, SNP genotyping was performed on a 100× dilution of the WGA samples. We leveraged an existing lion‐specific SNP panel, described in Bertola, Sogbohossou, et al. (2022) and Bertola, Vermaat, et al. (2022), consisting of 125 autosomal SNPs and 14 mtSNPs. To enhance local resolution, an additional 210 autosomal SNPs and 8 mtSNPs were added, resulting in a new, extended SNP panel of 335 autosomal SNPs and 22 mtSNPs. SNPs were selected based on the same criteria as outlined in Bertola, Sogbohossou, et al., 2022; Bertola, Vermaat, et al., 2022 with the addition that a preference was given to SNPs with a high minor allele frequency for increased power of individual identification. Genotyping was then carried out at the SNP genotyping facility of the Institute of Biology Leiden (IBL), Leiden University.

SNP genotyping was performed using an allele‐specific KASP technique (LGC Genomics) that relies on the competition of two allele‐specific forward primers and one common reverse primer, in combination with two fluorescently labelled reporter cassettes, containing a fluorophore and quencher. Each allele‐specific primer had a tail that was homologous to the tail of one of the two fluorophores of the reporter cassette. During PCR, the primer that did have the specific 3′‐end base was outcompeted by the allele‐specific primer. In addition, the complement sequence of the allele‐specific primer tail could bind with the complementary tail of the specific fluorophore. This then would emit a fluorescent signal upon excitation with which the SNP alleles could be determined (Biosearch Technologies, 2022). The results were analyzed in Kraken (LGC Genomics) and if required, necessary adjustments were made through manual inspection.

### 2.3 Data curation

After the genotyping process, we applied a quality control filter and excluded 30 samples with >20% missing data leaving a dataset of 141 samples. We then assigned these samples to meaningful population units, broadly based on the location where the samples were acquired and the breeding lion ranges as identified in the National Recovery and action plan for lions in Kenya 2020–2030. Out of the 141 samples, we excluded 19 additional individuals that could not be assigned to any population with at least 6 individuals, leaving a final dataset of 122 samples assigned to 12 populations as follows: Maasai Mara (*n* = 9), Shompole (*n* = 6), Nairobi (*n* = 12), Amboseli (*n* = 9), Chyulu Hills (*n* = 15), Tsavo (*n* = 8), Lake Nakuru NP—hereafter Nakuru (*n* = 9), Solio Ranch (*n* = 15), Nanyuki (*n* = 12), Laikipia (*n* = 9), Isiolo (*n* = 10) and Maralal (*n* = 8) (Table 1).

Although natural movement of lions has been documented between Tsavo West—Chyulu Hills and Amboseli NPs (Dolrenry et al., 2020), we separated samples from these sites to allow a more detailed look at these populations due to past lion translocations into Tsavo and previous findings of several monomorphic microsatellite loci in Amboseli (Bertola et al., 2015). The Solio Ranch lion population was also separated from Laikipia as it has been fenced for several years. Nanyuki was also treated as a population, because the samples were acquired from a repository and may have come from the wider Laikipia, Isiolo, Solio Ranch and/or from the north of Kenya, for example, Maralal.

### 2.4 Hardy Weinberg equilibrium (HWE) and genetic diversity

For each autosomal locus and population, HWE and respective *p*‐values were calculated using Arlequin version 3.5.2.2 (Excoffier & Lischer, 2010). We then tested each population for the number of loci that significantly differed from Hardy–Weinberg, while correcting for the number of monomorphic loci.

Allelic richness and private allelic richness were computed for each population following a rarefaction method to compensate for uneven sample sizes. We determined allelic richness (*A*_*R*_), private alleles (*A*_*P*_), inbreeding coefficients (*F*_IS_), expected (*H*_*E*_) and observed heterozygosity (*H*_*O*_) per population using the hierfstat package of RStudio version 4.2.0 (Goudet, 2005; R Core Team R, 2018).

### 2.5 Population structure and differentiation

To assess the distribution of mtDNA lineages in Kenya, a haplotype network was constructed using the 22 mtSNPs using Arlequin version 3.5.2.2 (Excoffier & Lischer, 2010). Isolation by Distance (IBD) was calculated by comparing pairwise *F*_ST_ to geographic distances using the Mantel Test in GenAlEx (Peakall & Smouse, 2012). Additionally, we ran Estimated Effective Migration Surfaces (EEMS) (Petkova et al., 2016) to assess the decay of genetic similarity in a geospatial context, by providing a matrix of genetic distances and GPS locations of samples. Kenya was divided into 500 spatial demes. We ran EEMS for 30 million iterations, using a burn‐in of 15 million, and doing three independent runs. Convergence was assessed visually and with the Gelman–Rubin diagnostic in the R package coda (Plummer et al., 2006).

An analysis of Molecular Variance (AMOVA) was performed on the autosomal SNPs and mtDNA to assess the variance between and within populations using Arlequin version 3.5.2.2 (Excoffier & Lischer, 2010). We then calculated the pairwise Fixation index (*F*_ST_) to assess genetic distance between populations.

STRUCTURE 2.3.4 (Pritchard et al., 2000) was used to assess population structure and was run using correlated allele frequencies. Ten runs were performed for *K* = 1 to *K* = 12, using 5,000,000 permutations and a burn‐in period of 500,000. The optimal *K*‐value was determined by Delta K in STRUCTURE Harvester (Earl & vonHoldt, 2012). CLUMPAK was used to merge the replications into a summarized barplot (Jakobsson & Rosenberg, 2007). We also estimated individual ancestry coefficients using Sparse Nonnegative Matrix Factorization algorithms (sNMF), implemented in the R package LEA (François, 2016) and we explored *K* values ranging from 1 to 12, each with 50 repetitions, and 4 values for the alpha regularization parameter (1, 10, 100 and 1000).

Clustering of individuals was further assessed using Principal Component Analysis (PCA) and Discriminant Analysis of Principal Components (DAPC) using the *adegenet* package of RStudio (Jombart, 2008). For DAPC, we utilized the xval function and retained 11 discriminant functions and preserved 67.8% of the genetic variation.

### 2.6 Fence and translocation data

To aid in interpreting results from the genetic analyses, we acquired lion translocation and fencing (i.e., management interventions) data from internal KWS unpublished reports, personal communication with wildlife managers, published papers and by use of a questionnaire. We sent out 20 questionnaires and received feedback from 16 wildlife managers in KWS parks and reserves, private and community conservancies. We obtained lion translocation data dating back to 2004. A lion was considered translocated if it was moved more than 100 km from where it was captured/resident (Jenkins, 1996).

We categorized lion populations in areas that were either partially or fully fenced as closed (i.e., with limited to no movement in and out; Nairobi, Nakuru and Solio Ranch) or open (there is known movement; remaining populations) (Table 1). Tsavo was categorized as open since the fenced sections do not limit lion movement. A parametric two sample *t*‐test was carried out in RStudio to test for significant genetic differences between closed and open lion populations using the estimates of *H*_*E*_, *A*_*R*_ and *F*_IS_.

## 3 RESULTS

All analyses were performed on the 122 samples, maintained after applying all quality filters (see ‘Methods’ section), and grouped into 12 populations (Figure 1). These samples had an average of 14% and a maximum of 17% missing data.

### 3.1 Genetic population structure

None of the loci consistently deviated from HWE for all 12 populations. High numbers of monomorphic loci were observed in the closed populations of Nakuru (53%) and Solio Ranch (31%) and in the open populations of Shompole (38%) and Amboseli (27%) (Table 1). Lions in both the unprotected Isiolo and protected areas of Tsavo had the highest allelic richness and expected heterozygosity scores. Higher observed heterozygosity scores were recorded in the protected populations of Tsavo and Laikipia. The inbreeding coefficient values ranged from −0.109 to 0.141 (from Nakuru to Isiolo). Nine private alleles were observed in the Isiolo lion population, one in Laikipia, Tsavo and Nairobi respectively (Table 1).

**TABLE 1:** Population, sample size (*N*), haplotypes detected (Haplo.), genetic diversity measures for 12 lion populations in Kenya.

Population	*N*	Haplo.	% monomorphic loci	% of loci out of HWE	*A*_*R*_	*A*_*P*_	*H*_*O*_	*H*_*E*_	*F*_IS_	% of translocated individuals received^a^	Status
Maasai Mara	9	1 and 2	20	6.0	1.31	0	0.33	0.31	−0.052 [−0.13, −0.02]	0	National reserve
Shompole	6	1	38	1.4	1.27	0	0.3	0.27	−0.106 [−0.15, 0.01]	0	Conservancy
Nairobi^b^	12	1 and 3	18	12.7	1.28	1	0.28	0.28	0.024 [−0.09, 0.04]	12.9%	National Park
Amboseli	9	1	27	6.6	1.26	0	0.26	0.26	−0.001 [−0.06,0.05]	0.9%	National Park
Chyulu Hills	15	1 and 4	18	10.9	1.31	0	0.32	0.31	−0.016 [−0.08, 0.01]	0	National park
Tsavo	8	1	15	4.2	1.34	1	0.33	0.34	0.015 [−0.04, 0.07]	40.5%	National park
Nakuru^b^	9	1	53	10.8	1.18	0	0.21	0.18	−0.109 [−0.22, 0.05]	1.7%	National park
Solio Ranch^b^	15	3	31	13.9	1.27	0	0.28	0.26	−0.017 [−0.13, 0.02]	0	Conservancy
Nanyuki	12	1 and 3	19	14.0	1.31	0	0.3	0.31	0.034 [−0.04, 0. 07]	0	NA^c^
Laikipia	9	3	19	5.9	1.32	1	0.33	0.32	−0.032 [−0.09, 0.02]	2.6%	Conservancies
Isiolo	10	3 and 5	13	4.4	1.34	9	0.3	0.34	0.141 [0.08, 0.18]	0	Both unprotected and protected national reserves
Maralal	8	3	18	3.6	1.33	0	0.32	0.33	0.012 [−0.04, 0.08]	0	Unprotected area

*Note*: Square brackets indicate 95% confidence limits. Per cent—of received individuals from translocation. Status‐protected area status.

Abbreviations: *A*_*P*_, private alleles; *A*_*R*_, allelic richness; *H*_*E*_, expected heterozygosity; *H*_*o*_, observed heterozygosity.

^a^ Individuals translocated to Meru (i.e., 41.4%) are not included in this table since the data for Meru was discarded due to >20% missing values.

^b^ Indicates the closed, that is, fenced populations.

^c^ The status of Nanyuki is not given as this is a repository.

**FIGURE 2:**
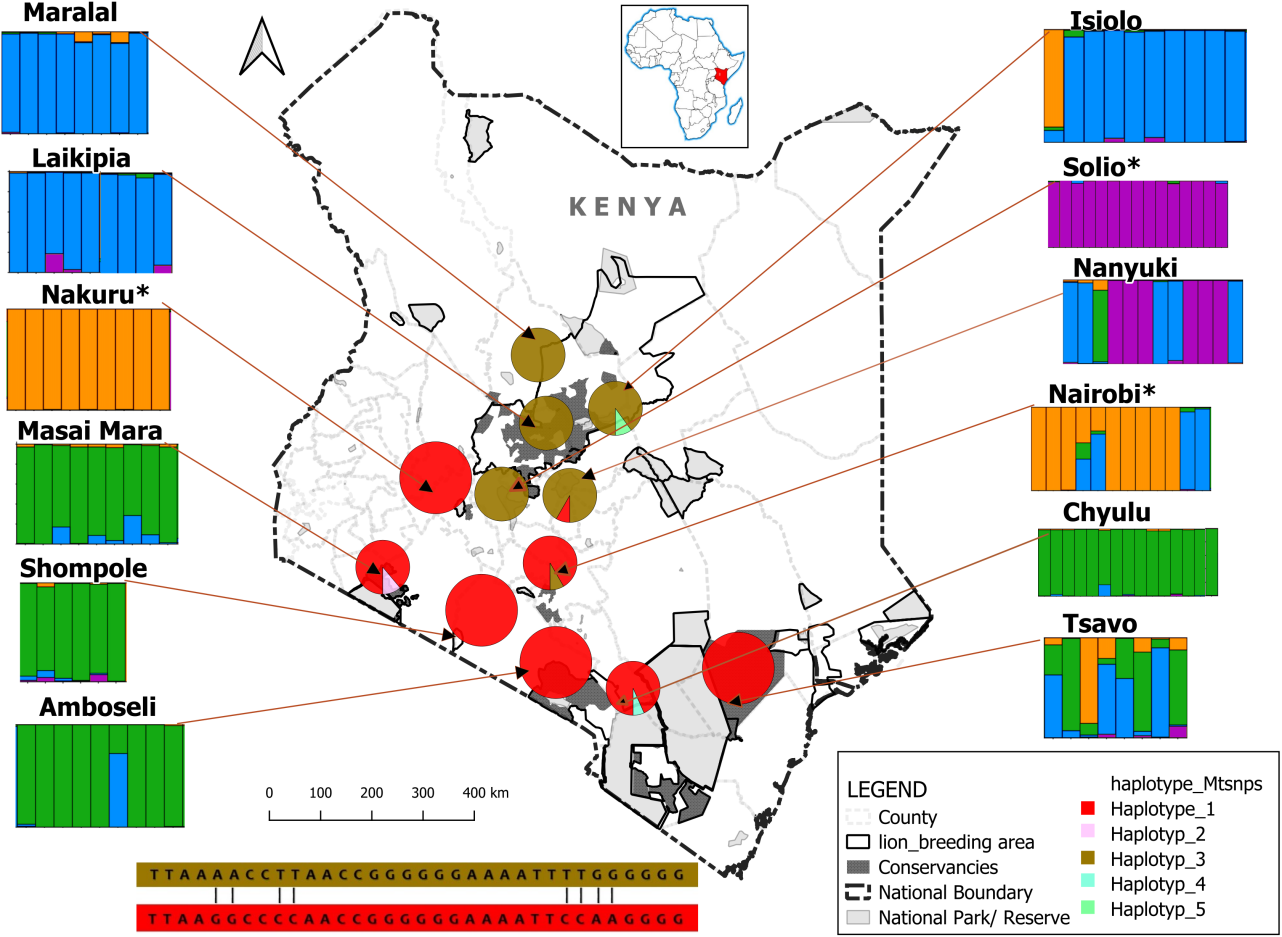
Barplots depicting STRUCTURE results for *K* = 4 based on 335 autosomal SNPs of 12 Kenyan lion populations. The pie charts show the 5 mtSNP haplotypes; the two major haplotypes differ by 8 nucleotides, as shown by the sequences below the map. Closed populations are marked with an *asterisk.

Using 22 mtSNPs, the presence of a total of 5 haplotypes was established, with 2 haplotypes being the most prevalent (haplotypes 1 and 3). The southern part of Kenya revealed the presence of 1 major haplotype, which differed by eight nucleotides from the northern part (Figure 2).

STRUCTURE analysis showed a DeltaK peak at *K* = 2 and *K* = 4 (Figure S1), indicating that two and four clusters best describe the underlying lion population structure in Kenya. Barplots revealed that at *K* = 2 all lion populations displayed substantial signs of admixture except for the closed Nakuru and Solio Ranch populations (Figure S1).

However, at *K* = 4, a geographic population structure of lions between northern + middle and southern Kenya was revealed except for Tsavo (barplots Figure 2) which exhibited signs of admixture. sNMF revealed similar results indicating a split between the northern (green) and southern (yellow) lion populations and highlighted the closed populations, that is, Nairobi and Nakuru were grouped together (pink), while Solio formed a distinct cluster (blue), and the Tsavo population was on the intersection of both green and yellow clusters indicating that the population is possibly admixed (Figure S2).

**FIGURE 3:**
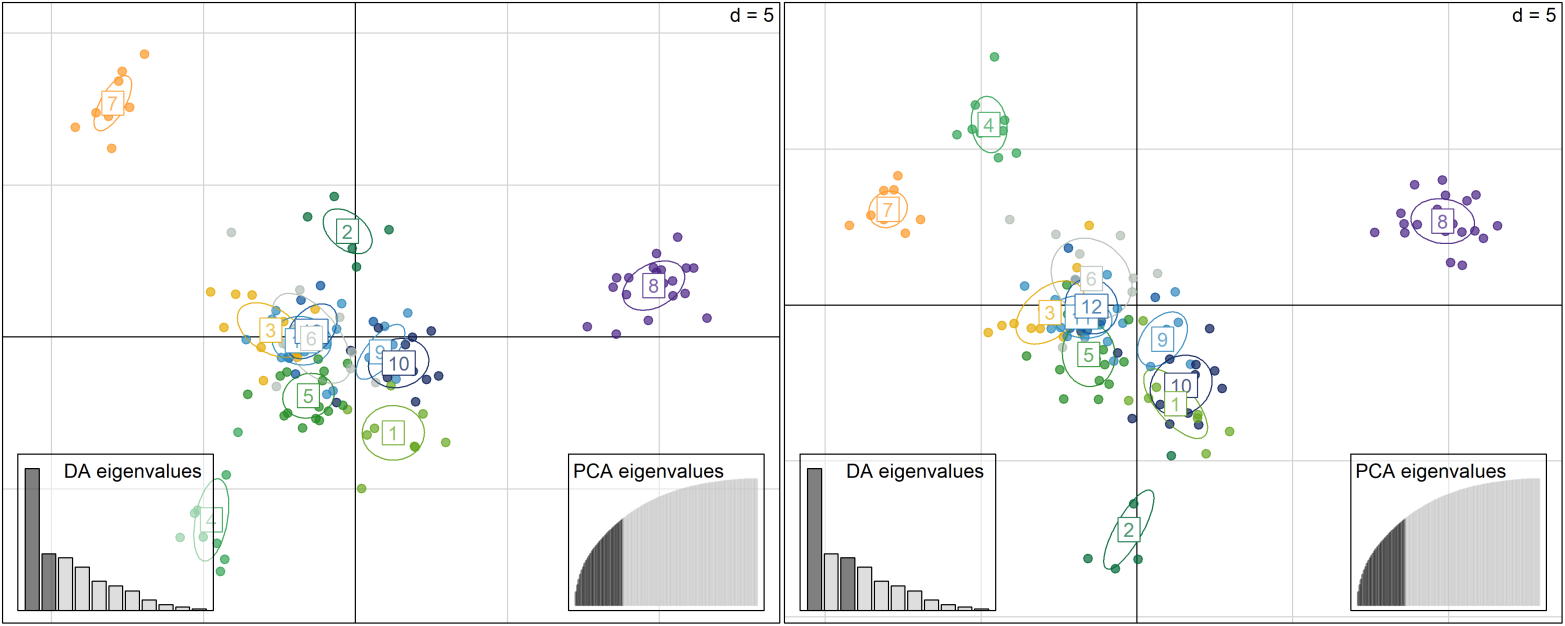
DAPC based on 335 autosomal loci of 12 Kenyan lion populations showing results for axes 1 and 2, 1 and 3. DAPC was performed with 11 retained discriminant functions, preserving 67.8% of the genetic variation. The numbers correspond to 1—Maasai Mara; 2—Shompole, 3—Nairobi, 4—Amboseli, 5—Chyulu Hills, 6—Tsavo, 7—Nakuru, 8—Solio Ranch, 9—Nanyuki, 10—Laikipia, 11—Isiolo and 12—Maralal.

PCA (Figure S3), revealed clustering of Solio Ranch, Nakuru and Nairobi for PCA 1 and 2 while PCA 3 and 4 also revealed a clustering for Amboseli and Shompole. The DAPC results showed a similar clustering, again highlighting Solio Ranch and Nakuru, but also showing strong clustering of Amboseli and Shompole (Figure 3).

### 3.2 Genetic differentiation

We found a weak negative correlation between genetic distance and geographic distance, but we did not find a statistically significant IBD pattern, that is, IBD (Mantel *r* = 0.07, *p* = 0.08). EEMS analysis also indicated that the connectivity between populations was quite low, especially for the closed populations of Nakuru and Solio Ranch that were surrounded by very low mrate values (Figure S4).

AMOVA results for the autosomal SNPs revealed that 14% of the total variation was found between populations and the remaining 86% was within populations. On the other hand, the mtDNA markers indicated that 38% of the variation was between populations and 62% within populations (Table S1).

All populations displayed pairwise *F*_ST_ values significantly different from zero except Isiolo and Maralal. Pairwise *F*_ST_ values of closed Nakuru and Solio Ranch populations showed a distinctive pattern and were more distant from the other populations and from each other (Table 2).

**TABLE 2:** Pairwise *F*_ST_ values for the 12 Kenyan lion populations based on 335 autosomal SNPs.

	Maasai mara	Shompole	Nairobi	Amboseli	Chyulu hills	Tsavo	Nakuru	Solio ranch	Nanyuki	Laikipia	Isiolo	Maralal
Maasai mara	–											
Shompole	0.123	–										
Nairobi	0.156	**0.236**	–									
Amboseli	0.148	**0.232**	**0.236**	–								
Chyulu Hills	0.087	0.120	0.155	0.141	–							
Tsavo	0.040	0.100	0.104	0.105	0.033	–						
Nakuru	**0.273**	**0.310**	**0.203**	**0.347**	**0.263**	0.182	–					
Solio ranch	**0.203**	**0.226**	**0.265**	**0.248**	0.161	0.123	**0.359**	–				
Nanyuki	0.117	0.146	0.180	0.177	0.097	0.057	**0.273**	0.037	–			
Laikipia	0.115	0.147	0.165	0.196	0.087	0.061	**0.286**	0.118	0.036	–		
Isiolo	0.080	0.127	0.138	0.163	0.074	0.018	**0.257**	0.115	0.050	0.021	–	
Maralal	0.076	0.121	0.128	0.154	0.077	0.021	**0.249**	0.105	0.039	0.018	−0.013	–

*Note*: Bold values (>0.2) indicate stronger genetic differentiation between populations.

### 3.3 Fence and lion translocations

The parametric two‐sample *t*‐tests revealed that there were no statistically significant differences in genetic variability between the open and closed populations, that is, *H*_*E*_ (*t* = 2.1894, *p*‐value = 0.1388); *A*_*R*_ (*t* = 2.0317, *p*‐value = 0.1584) and *F*_IS_ (*t* = 0.73776, *p*‐value = 0.5078).

**FIGURE 4:**
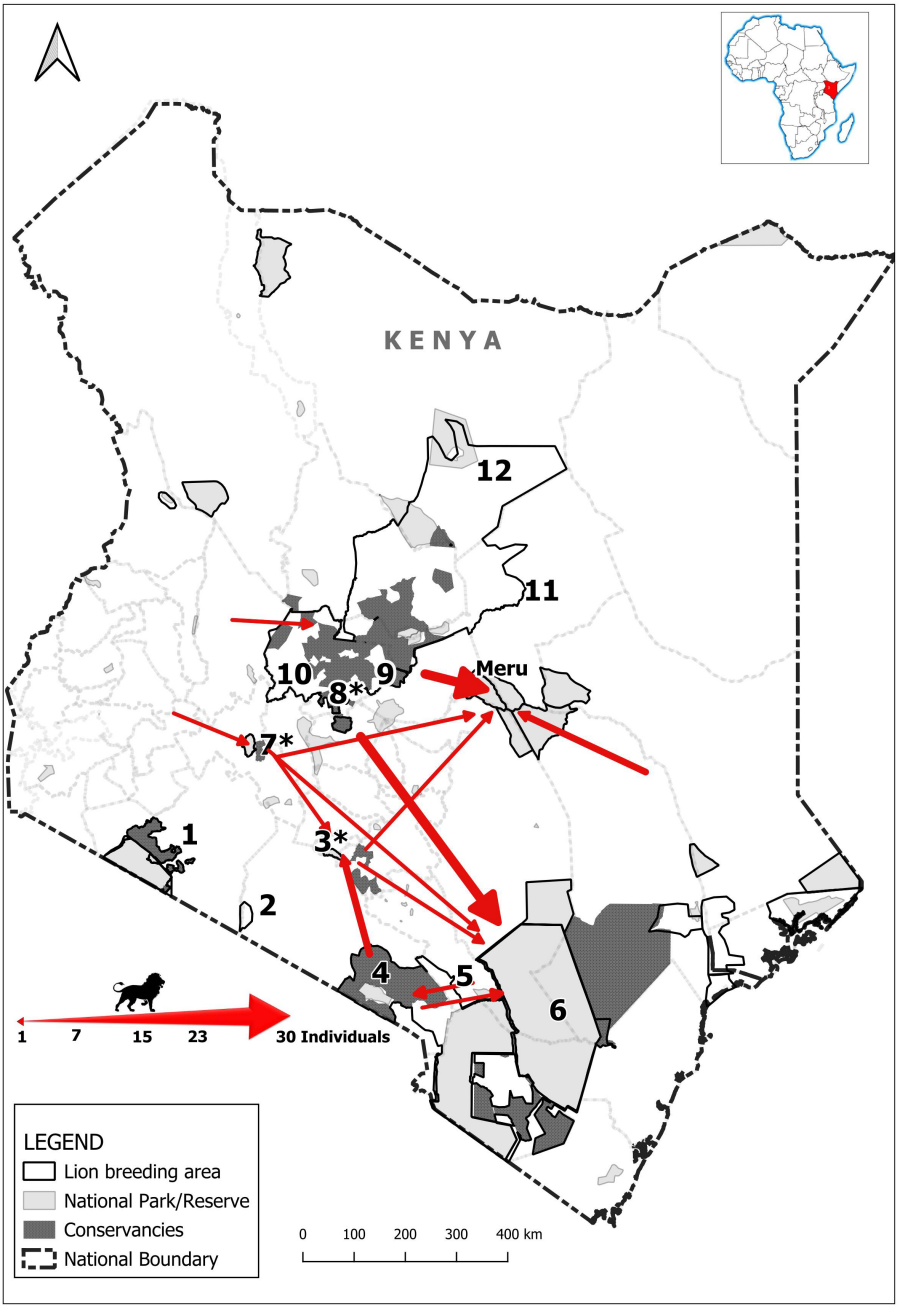
Lion translocation events 2004–2020 are illustrated by arrows. Closed populations are marked with an *asterisk. The width of the arrows reflects the number of individuals translocated to an area. The numbers correspond to 1—Maasai Mara; 2—Shompole, 3—Nairobi, 4—Amboseli, 5—Chyulu Hills, 6—Tsavo, 7—Nakuru, 8—Solio, 9—Nanyuki, 10—Laikipia, 11—Isiolo and 12—Maralal.

Figure 4 illustrates lion translocation events from 2004 to 2020, with Tsavo and Meru NPs being the main recipients. During this period a total of 116 individuals were translocated: 48 to Meru (13 adult and 2 sub‐adult males, 10 adult females and 23 unsexed/unaged individuals); 47 to Tsavo (9 adult and 12 sub‐adult males, 16 adult and 6 sub‐adult females and 4 unsexed sub‐adults) and the remaining 21 were taken to areas such as Amboseli and Nairobi among others (4 adult and 3 sub‐adult males, 3 adult and 1 sub‐adult female, 7 cubs, 3 unsexed and unaged individuals).

## 4 DISCUSSION

### 4.1 Genetic diversity of lions in Kenya

Building upon the prior research by Bertola (2015) and Bertola et al. (2015) and Bertola, Sogbohossou, et al. (2022) and Bertola, Vermaat, et al. (2022) that covered the lion's entire range in Africa and Asia, our study provides the first comprehensive analysis of the genetic composition of Kenya's lion population. The AMOVA analysis revealed that the predominant portion of genetic variation was within individual populations rather than between populations, signifying substantial genetic diversity within each distinct population. Additionally, we established the presence of two major haplotypes, one from the south and the other from the north parts of Kenya as previously reported (Bertola et al., 2015). These haplotypes point to different genetic lineages, likely as a result of large‐scale environmental changes caused by climatic fluctuations of the Pleistocene epoch. During this time, several species ranges were confined to isolated refugia in response to shifts in vegetation and habitat, leading to differentiating lineages (Bertola et al., 2016). These lineages are also believed to have been brought about by anthropogenic activities that resulted in habitat fragmentation and subsequent geographic isolation leading to differentiation (Creel et al., 2019; Curry et al., 2020). Therefore, for the lion population in Tsavo, while we attribute admixture to historical translocations (discussed in Section 4.3) we also attribute it to Tsavo being a possible natural suture zone where species of different genetic lineages co‐occur as a result of environmental conditions or habitat transformation that allowed secondary contact among diverged lineages (Lorenzen et al., 2008, 2012). Tsavo NP covers a large area of ∼48,300 km^2^ and is situated between the Horn of Africa (occupied by the northern haplotype) and the Maasai Steppe—encompassing southern Kenya and north Tanzania (southern haplotype). The Galana River within Tsavo or Athi River further north might have served as the historical boundary between the two haplotypes. Then, habitat transformation brought about by human activities and elephants may have altered these boundaries, potentially allowing genetically distinct populations to come into contact and admix (Lorenzen et al., 2008). Also, the presence of other genetically divergent species such as the desert (*Phacochoerus aethiopicus*) and common (*P*. *africanus*) warthogs (Garcia‐Erill et al., 2022); Somali (*Struthio molybdophanes*) and Maasai (*S*. *camelus*) ostrich (Birdlife International, 2018); Peter's gazelle (*N*. *petersii*) and Grant's (*Nanger granti*) (Garcia‐Erill et al., 2021; Lorenzen et al., 2008) support this possibility.

Additionally, we attribute the high genetic diversity indices observed in Tsavo NP to translocation and to the presence of a large lion population whose movement is generally unrestricted, with proven genetic exchanges with the lion populations in Chyulu Hills and Amboseli NPs (Dolrenry et al., 2020). This unrestricted movement was confirmed by the low pairwise *F*_ST_ values ranging from 0.03 and 0.1 between Tsavo and the protected areas of Chyulu Hills and Amboseli.

As with the microsatellite data published by Bertola et al. (2015), we found a significant proportion of monomorphic loci in the Amboseli lion population. This population had previously experienced a strong bottleneck with subsequent immigration (Dolrenry et al., 2014). Despite movement of lions from the neighbouring Chyulu Hills and Tsavo, the population still had low observed heterozygosity and diminished allelic richness. Indicating that, while the population numbers rebounded, after the strong bottleneck in the 1990s, recovery of genetic diversity is much harder to achieve and may need management interventions such as genetic reinforcement.

The lion population in Shompole occupies the protected areas of Shompole (620 km^2^) and Olkiramatian conservancies (270 km^2^); with the exception of the Rift Valley escarpment to the east, the conservancies are part of a contiguous transboundary ecosystem encompassing >8000 km^2^ across southern Kenya and northern Tanzania (Schuette et al., 2013). Adjacent lion populations include Torosei and Musenge (~30–50 km east), Naimina Enkiyio forest ~25 km west of Shompole and at landscape level Amboseli NP and the Maasai Mara National Reserve (Western et al., 2022). While lions are known to traverse large distances, we found a rather high percentage (38%) of monomorphic loci and a distinct clustering in the PCA and DAPC. Therefore we can presume that either our dataset was not sufficient/representative of the lions in Shompole or that movement of lions across the landscape is limited by a natural barrier, that is, the Rift Valley escarpment. It is also possible that the increasing land subdivision and farmland production in surrounding rangelands may be limiting lion movement (Dolrenry et al., 2014; Schuette et al., 2013).

The significant diversity scores within the Laikipia lion population could reflect potential immigration from neighbouring populations including Maralal and Isiolo, following the historical decline in Laikipia in the 1900s. Possible immigration of lions into Laikipia from Mararal and Isiolo is supported by the low genetic distance between these three populations. As mentioned, Maralal and parts of Isiolo host lion populations that mostly occur in areas without formal protection, they exhibited high levels of genetic diversity comparable to protected areas of Tsavo and Maasai Mara, highlighting the importance of lion populations in unprotected areas and their potential contribution to the larger gene pool. However, it is important to note that movement of lions in Isiolo has increasingly become restricted due to development activities and retaliatory killings (Bhalla, 2017). The impact of these activities can be seen by the presence of nine private alleles, an indication of low gene flow since increased migration lowers the proportion of private alleles (Curry et al., 2019). Additionally, the *F*_IS_ value indicated an excess of homozygotes which may also be attributed to a small population size.

### 4.2 Impact of fencing

The Nakuru population resides within a small NP that is approximately 188 km^2^ and has been enclosed by an electric fence for about four decades. This population was established in the mid‐1980s and early 1990s through the introduction of six individuals from Nairobi, Aberdare and Tsavo NPs. Since then, there have been no known additional introductions or immigration events. Even though there is occasionally intermixing with the only neighbouring population from Soysambu Conservancy as a result of occasional fence breaches, both populations originated from the same founder population, making the population closed and isolated (Elliot et al., 2020). Likewise, the Solio Ranch population inhabits a compact area ~161 Km^2^ and until 2019 the fence had been effective in preventing lion movement for nearly five decades. While, the Nairobi NP population resides in a small NP (~117 km^2^), sections of the park bordering urban areas are fenced but movement along the unfenced southern boundary is restricted by human settlements (Lesilau et al., 2021).

Nakuru NP and Solio ranch displayed the lowest overall genetic diversity indices compared to the other populations. While there was no evidence of inbreeding based on the *F*_IS_ results, distinct genetic clusters were observed in the PCA and DAPC analysis plots, as well as in the sNMF and the structure bar plots at *K* = 2 and *K* = 4, indicating potential genetic erosion. Additionally, EEMs revealed a stronger decay of genetic similarity than expected under isolation by distance, reflecting reduced gene flow and population isolation. Further, the comparatively higher *F*_ST_ values suggest limited a gene flow and stronger genetic differentiation. Collectively, these findings suggest significant genetic structuring and potential isolation for Nakuru and Solio Ranch lion populations. Considering their limited genetic diversity and likely limited capacity to adapt to future environmental changes, they face an elevated risk of decline and potential extinction if no intervention is made (Björklund, 2003; Mathur et al., 2023).

The Nairobi NP lion population exhibited average genetic diversity and formed a diffuse cluster on PCA 1 and 2; with sNMF, it was grouped together with the Nakuru population (Figure S2). We interpret this as an indication of an ongoing genetic drift due to limited gene flow. Although Dolrenry et al., 2020, recorded the movement of an individual from Amboseli to Kapiti plains that form the dispersal areas south of Nairobi NP, the frequency of lion movement between Amboseli and Nairobi is mostly unknown and may be infrequent.

### 4.3 Translocation

Translocation of ‘problem’ lions in Kenya has primarily been used as a conflict mitigation tool. This strategy dates back to the mid‐1950s, with Tsavo and Meru serving as the main recipient sites due to their vastness and presumed large prey base (Jenkins, 1996). However, lion and other carnivore translocations have often been subject to debate, with successful cases being attributed to translocation into unfenced areas, selection of younger individuals, and the method of release (i.e., soft) (Thomas et al., 2023). On the other hand, failure has been attributed to significant mortality rates among the translocated individuals due to capture‐related stress, extensive post release movements or being killed by conspecifics (Linnell et al., 1997; Morapedi et al., 2021). More so, translocations aimed at solving human–lion conflicts have generally been reported to fail (Fischer & Lindenmayer, 2000).

The likelihood of translocated individuals surviving in Tsavo may be impeded by the presence of an abundant resident population (~459 individuals, Kenya Wildlife Service, 2020) and the presence of a relatively large number of nomadic males already in the park (Kays & Patterson, 2002). Nevertheless, admixture from past translocations into the area is apparent in its genetics. This is further supported by the low *F*_ST_ values between Tsavo and all the other populations Table 2.

In Meru NP, Goeminne (2019) gave an account of a ‘problem’ male lion translocated in 2017 that successfully integrated with a resident pride and was able to breed. We however could not confirm admixture for the Meru population since its samples had >20% missing values and were removed from the analysis.

## 5 CONCLUSION AND RECOMMENDATIONS

Understanding existing patterns of genetic diversity, combined with information on both historical and contemporary connectivity between populations, forms the baseline for effective genetic management. It allows managers and policymakers to make evidence‐based decisions regarding either restoring connectivity and gene flow between populations or separately managing populations with unique evolutionary histories. Hereby, patterns of genetic variation can be optimally conserved, while simultaneously acknowledging challenges such as limited funds, which may result in the need for prioritization.

Genetic guidelines detailed by Miller et al., 2013, Bertola et al., 2021 and Becker et al., 2022 are relevant for the closed Nakuru NP and Solio Ranch populations where genetic erosion was detected. For example, if in the future there would be a decision to supplement these populations; Solio Ranch should be supplemented by individuals from the north. The situation in Lake Nakuru NP is more complex, as the population was founded by individuals from the south (Nairobi and Tsavo) and north (Aberdares NP); this population would probably benefit from restocking with individuals from the south since this would likely have been the original genetic signature. These recommendations align with those outlined by Bertola, Sogbohossou, et al., 2022and Bertola, Vermaat, et al. (2022), offering genetic management guidance for policymakers by providing a suitability matrix for source and target populations along with a decision tree to guide lion translocations.

For the partially fenced Nairobi population, we recommend efforts to try to re‐establish corridors that will allow and enhance movements, between Nairobi‐Kapiti plains and Amboseli. Collaring using GPS or satellite collars, especially males of dispersal age, can aid in the identification of natural corridors. However, in view of how long this process may take, if successful, the Nairobi population could benefit from human‐mediated dispersal through translocation from southern populations as a medium‐term goal. However, if translocation is to be used as a genetic restoration tool, we recommend following the guidelines detailed by Miller et al. (2013). In addition to the use of adaptive management techniques, implementation of long‐term monitoring is crucial, as translocated individuals may present management challenges, particularly stemming from conflicts with humans and resident lion populations (Abell et al., 2013; Morapedi et al., 2021).

Establishment of lion conservation units that cover both protected and unprotected areas to allow connectivity between populations would be crucial for the open Shompole, Maralal and Isiolo populations as well as the previously extirpated populations of Amboseli and Laikipia. This would entail a focus on genetic restoration efforts through the maintenance of suitable habitats that allow movement between populations and, whilst doing so, provide a sufficient prey base. Enhancing human tolerance to allow the movement of even a few individuals through intervening habitats can have a significant impact on lion conservation by facilitating genetic exchange between populations (Dolrenry et al., 2014). Therefore, mitigation of human‐lion conflicts and engaging communities in the conservation process is fundamental.

For the admixed Tsavo NP (and possibly Meru NP) lion populations, to understand the effects of admixture, we recommend establishing long‐term monitoring programs for population and social dynamics including genetic analyses for admixture assessment to inform conservation decisions and actions. In the meantime, wildlife managers should heed the recommendations of Bertola, Sogbohossou, et al. (2022) and Bertola, Vermaat, et al. (2022) on separately managing populations with unique evolutionary histories.

We recommend the integration of our results into the lion translocation protocol of KWS and recommend that it takes into account the genetic characteristics of both translocated and recipient lion populations, besides factors such as densities and sex ratios of conspecifics at recipient sites, target individuals and availability of wild prey. This integration of genetics into translocation policy should also consider evolutionary histories and distinguish between translocation for population restoration, for management of ‘problem’ individuals and possibly translocation of ‘problem’ individuals with an aim to restore genetic diversity.

The outcomes of this study provide the scientific data required to integrate genomics into wildlife management and policy in Kenya. This is also applicable to other lion range countries and for other species.

## CONFLICT OF INTEREST STATEMENT

The authors declare that they have no known competing financial interests or personal relationships that could have appeared to influence the work reported in this paper.

## Supporting information


Figure S1.

Figure S2.

Figure S3.

Figure S4.

Table S1.

## Data Availability

Data for this study are available at: 10.5061/dryad.s4mw6m9d8.
