# Supplementary material for: Genetic diversity of lion populations in Kenya: Evaluating past management practices and recommendations for future conservation actions
Source: Evol Appl. 2024 Mar 19;17(3):e13676. doi: 10.1111/eva.13676 (PMC10950092; doi:10.1111/eva.13676)
Supplement: Supplementary file 1 — Figure S1. [file EVA-17-e13676-s002.doc]

**Supplementary figures**


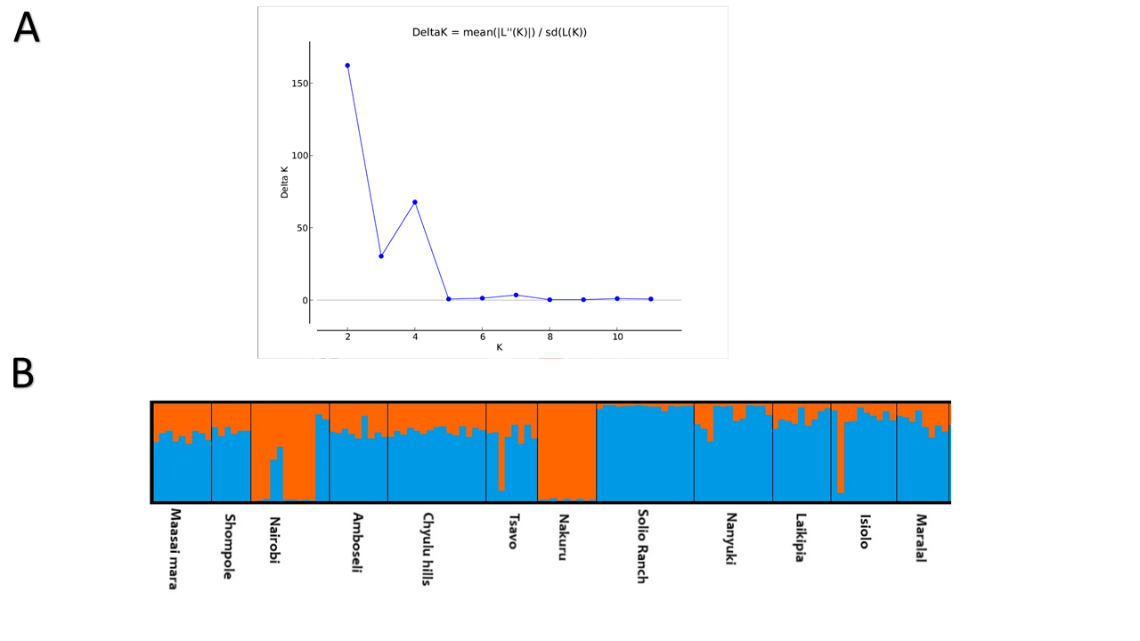


**Supplementary Figure 1. A. DeltaK analysis obtained from STRUCTURE harvester showing a peak at K = 2 and K = 4. B. Assignment values based on the STRUCTURE run for K =2 based on 335 autosomal SNPs for the 12 Kenyan lion populations.**
