# Supplementary figures and images for: Genetic diversity of lion populations in Kenya: Evaluating past management practices and recommendations for future conservation actions
Source: Evol Appl. 2024 Mar 19;17(3):e13676. doi: 10.1111/eva.13676 (PMC10950092; doi:10.1111/eva.13676)

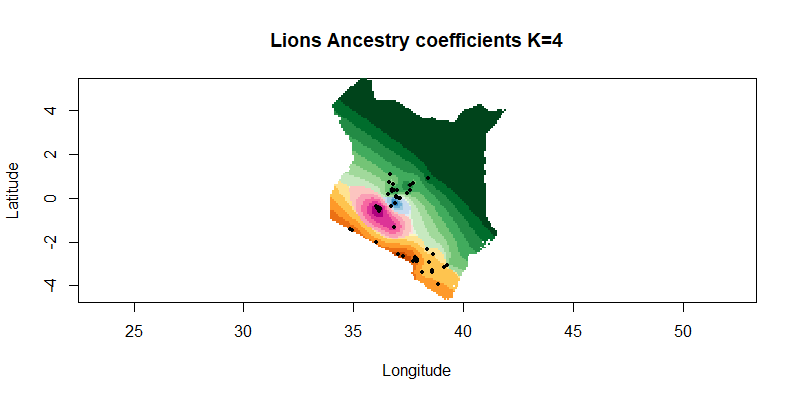

Supplement: Supplementary file 2 — Figure S2. [file EVA-17-e13676-s005.png]

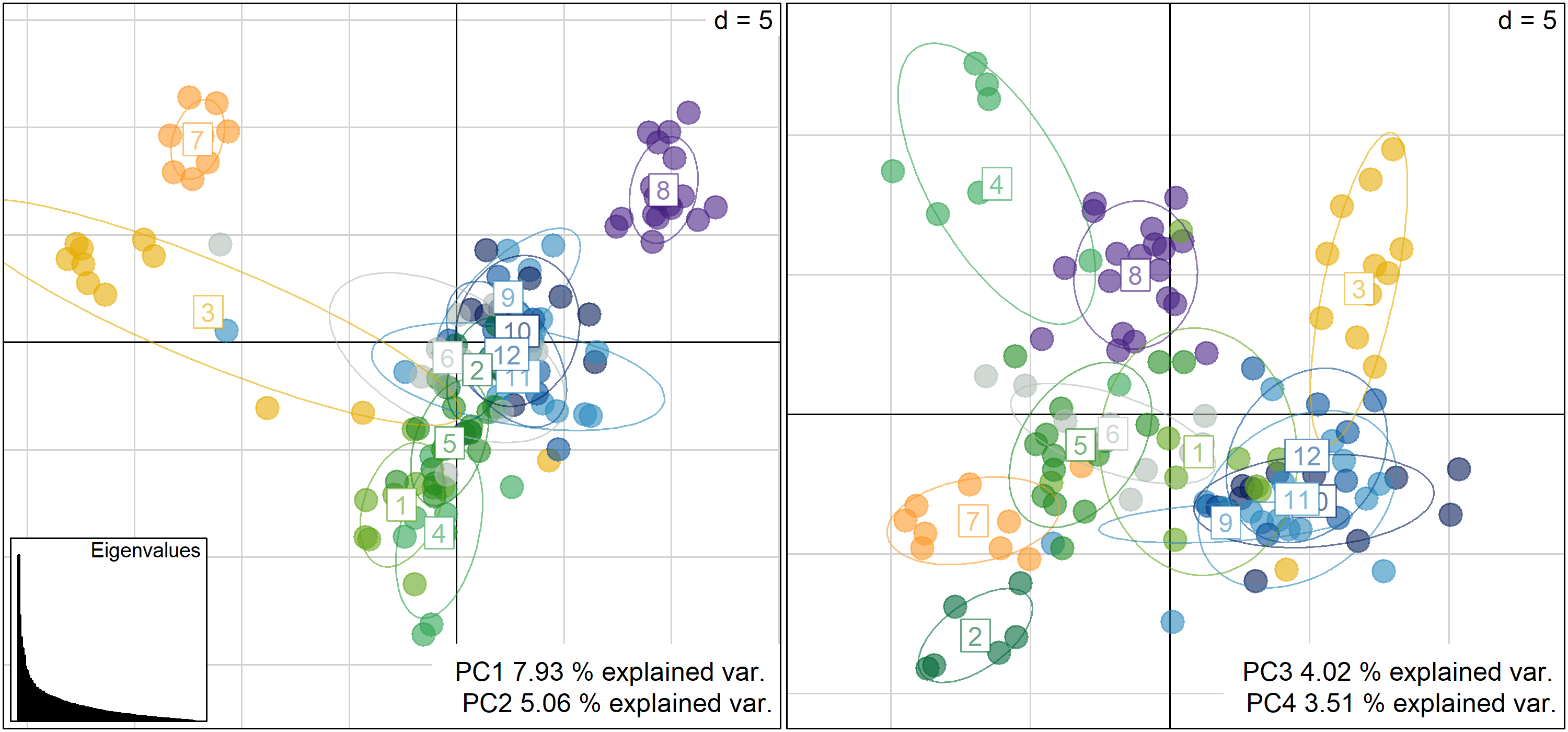

Supplement: Supplementary file 3 — Figure S3. [file EVA-17-e13676-s004.jpeg]

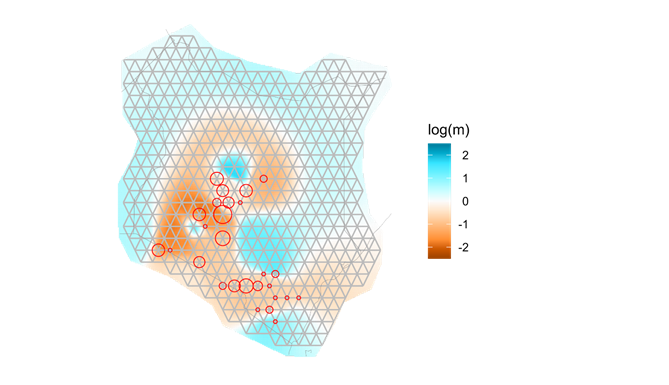

Supplement: Supplementary file 4 — Figure S4. [file EVA-17-e13676-s001.png]
