# Supplementary material for: Genetic diversity of lion populations in Kenya: Evaluating past management practices and recommendations for future conservation actions
Source: Evol Appl. 2024 Mar 19;17(3):e13676. doi: 10.1111/eva.13676 (PMC10950092; doi:10.1111/eva.13676)
Supplement: Supplementary file 5 — Table S1. [file EVA-17-e13676-s003.docx]

**Supplementary Table 1. AMOVA results for 122 lions from 12 populations in Kenya. The significance of differentiation within and among populations was tested by 999 permutations**

| **MtDNA** | | | | | | |
| --- | --- | --- | --- | --- | --- | --- |
| **Source** | **Degrees of Freedom** | **Sum of Squares** | **Mean Sums of Squares** | **Estimated Variance** | **Percentage of Variation** | **p-value** |
| Among Populations | 11 | 450.216 | 40.929 | 3.464 | 38% | 0.001 |
| Within Populations | 111 | 617.313 | 5.561 | 5.561 | 62% | 0.001 |
| Total | 122 | 1067.528 |  | 9.025 | 100% |  |
| **Autosomal SNPs** | | | | | | |
| Among Populations | 11 | 2869.363 | 260.851 | 9.867 | 14% | 0.001 |
| Within Populations | 232 | 14263.781 | 61.482 | 61.482 | 86% | 0.001 |
| Total | 243 | 17133.143 |  | 71.349 | 100% |  |
